# *Cannabis* for Medical Use: Analysis of Recent Clinical Trials in View of Current Legislation

**DOI:** 10.3389/fphar.2022.888903

**Published:** 2022-05-25

**Authors:** F. Baratta, I. Pignata, L. Ravetto Enri, P. Brusa

**Affiliations:** Department of Drug Science and Technology, University of Turin, Turin, Italy

**Keywords:** medical *Cannabis*, clinical trials, study protocols, legislation, law

## Abstract

*Cannabis* has long been regarded as a recreational substance in the Western world. The recent marketing authorization of some medicinal products of industrial origin and the introduction onto the market of inflorescences for medical use mean that medical doctors can now prescribe *Cannabis*-based medicines in those countries which allow it. Nevertheless, there is still considerable controversy on this topic in the scientific community. In particular, this controversy concerns: the plant species to be used; the pathologies that can be treated and consequently the efficacy and safety of use; the routes of administration; the methods of preparation; the type and dosage of cannabinoids to be used; and, the active molecules of interest. As such, although medical *Cannabis* has been historically used, the results of currently completed and internationally published studies are inconclusive and often discordant. In light of these considerations, the aim of this work is to analyse the current legislation in countries that allow the use of medical *Cannabis*, in relation to the impact that this legislation has had on clinical trials. First of all, a literature search has been performed (PubMed and SciFinder) on clinical trials which involved the administration of *Cannabis* for medical use over the last 3 years. Of the numerous studies extrapolated from the literature, only about 43 reported data on clinical trials on medical *Cannabis*, with these mainly being performed in Australia, Brazil, Canada, Denmark, Germany, Israel, Netherlands, Switzerland, the United Kingdom and the United States of America. Once the reference countries were identified, an evaluation of the legislation in relation to *Cannabis* for medical use in each was carried out via the consultation of the pertinent scientific literature, but also of official government documentation and that of local regulatory authorities. This analysis provided us with an overview of the different legislation in these countries and, consequently, allowed us to analyse, with greater awareness, the results of the clinical trials published in the last 3 years in order to obtain general interest indications in the prosecution of scientific research in this area.

## 1 Introduction

*Cannabis* was widely used in the past for its curative properties. The earliest records of its medicinal use date back to China where *Cannabis* has been cultivated for millennia for use as a fiber, food, and medicine. Over time, it spread to the whole of Asia, the Middle East, and Africa. In the West, the plant started to attract scientific interest only in the 20th century. However, in the last century, the cultivation, sale, and use of *Cannabis* was made illegal in the majority of countries (Lafaye, et al., 2017; Pisanti and Bifulco, 2019; Romano and Hazekamp, 2019; Arias, et al., 2021).

In the last few decades, there has been revived support for its decriminalisation, and legalisation for medical uses thanks to new and scientifically founded indications of its potential therapeutic value. This is partly due to the support gained in the media, and to the high expectations for its efficacy, even though these hopes, for many diseases, are not sufficiently supported by scientific research (Hill, 2015; Whiting, et al., 2015).

The phytocomplex of *Cannabis* plants is made up of more than 500 molecules, of which about a hundred belong to the Cannabinoid chemical class. Among these molecules, even small variations in molecular structure can produce significantly different effects. The molecules of greatest interest to pharmacologists are the decarboxylated forms of 9-tetrahydracannabinol (THC) and cannabidiol since these are easily absorbed in the intestine (Grotenhermen, 2003; Gould, 2015; Baratta, et al., 2019; Baratta, et al., 2021).

Recently, *Cannabis* based industrial medicines have been approved for sale, and medical use inflorescences have been made available. This has given medical doctors, in those countries which allow it, the option to prescribe *Cannabis*-based products. At present, the most widely available products are: Marinol^®^ (AbbVie Inc) and Syndros^®^ (Benuvia Therapeutics) which contain dronabinol, an isomer of delta-9-tetrahydrocannabinol; Cesamet^®^ based on nabilone (Meda Pharmaceuticals Inc.), another synthetic cannabinoid; Sativex^®^ (GW Pharma Ltd.), based on an ethanol extraction of *Cannabis sativa*; and Epidiolex^®^ ^1^ (Greenwich Biosciences), which contains CBD (Casiraghi, et al., 2018).

A variety of pharmaceutical-grade inflorescence products are also available on the market. Usually, the label only indicates the concentrations of THC and CBD. This is a critical point as the phytocomplex of medical *Cannabis* contains many active molecules which contribute to the “Entourage effect,” a hypothesis postulating a positive synergic action between cannabinoids and terpenes (Stella, et al., 2021; Baratta, et al., 2022).

Given the increasing availability of the above products, many countries have introduced specific legislation, regulations, and guidelines regarding the use of medical use *Cannabis* in the treatment of various pathologies. Nevertheless, debate continues around this subject within the scientific community. The main points of contention are the correct plant varieties to be used, the pathologies to be treated, and, consequently, the efficacy and safety of their use. There are no universally shared indications on the optimum administration route, the preparation methodology, the definitive types of cannabinoids and dosages to recommend, or even the identity of the active molecule of interest. This controversy stems in large part from the findings of the clinical trials conducted till now. Although the number of studies and publications is growing rapidly, for many diseases the results are often contradictory or inconclusive. All too often, these trials were performed on a non-homogeneous population, and utilising diverse plant material, extraction methods, dosages, pharmaceutical forms, and administration routes. Moreover, the trials were often conducted without a control group (Stella, et al., 2021).

In light of all these considerations, the objective of this work is to analyse the current legislation and regulations in a number of countries where medical use *Cannabis* is permitted in order to evaluate any relationship of these on the design of clinical trials carried out there.

## 2 Materials and Methods

We carried out a literature search (PubMed and SciFinder) for clinical trials with medical *Cannabis* published in the last 3 years (2019/01/01–2021/12/15). We excluded literature reviews, non-clinical trials, and articles about non-medical use *Cannabis*. We also considered published articles about clinical trial protocols to be carried out. The key search terms used were clinical trials, medical *Cannabis*, and medical use.

After the publications had been selected, the countries of origin were identified in order to perform an evaluation of the current regulations in each regarding medical *Cannabis*. The scientific literature, and relevant official publications from government and local authorities were consulted for this analysis.

Finally, the characteristics and the results of the clinical studies were analysed to evaluate any possible link to the state legislation where the studies had been carried out.

## 3 Results

Of the 400 matches from the literature search, only 10% (43) of the publications reported data from trials or clinical protocols regarding medical *Cannabis*. The relevant trials were carried out in: Australia, Brazil, Canada, Denmark, Germany, Israel, Netherlands, Switzerland, the United Kingdom, and the United States of America. Given their geographical distribution, these countries can be considered of interest despite the small number of studies available.

For each of the countries in question, the current legislation on medical *Cannabis* was analysed, and some specific features are reported such as: prescription procedure, indicated pathologies for medical *Cannabis*, products available for sale, dispensation forms, authorisation to grow *Cannabis* for medical use, and reimbursement procedure.

### 3.1 Current Legislation

#### 3.1.1 Australia

Although there are some regulatory differences among the federal states regarding the importation of products, and the qualification required to write a prescription, medical *Cannabis* may be prescribed after receiving authorisation from the Therapeutic Goods Administration, through the Special Access Scheme for an individual patient, or through the Authorized Prescriber Scheme for a group of patients with the same condition. Products of industrial origin are exempt from these schemes as approval for sale has already been granted (Sativex^®^ and Epidiolex^®^).

As well as Sativex^®^ and Epidiolex^®^, indicated for the treatment of spasticity in multiple sclerosis and paediatric epilepsy, herbal- *Cannabis* based products may also be prescribed. The most common conditions are spasticity in multiple sclerosis, nausea or vomiting caused by anti-tumoral chemotherapy, pain or anxiety in patients with terminal diseases, and refractory child epilepsy. The physician may in any case write a prescription for pathologies other than those indicated.

Pharmacies are authorised to dispense medical *Cannabis*-based products.

The cost of the therapy is not subsidised by the government.

Alcohol and Drug Foundation, 2021; Australian Capital Territory Government, 2021; Australian Government, 2017a; Australian Government, 2017b; Australian Government, 2018; Australian Government, 2020; Australian Government, 2021; Australian Institute of Health and Welfare, 2019; Castle, et al., 2019; Centre for Medicinal Cannabis Research and Innovation, 2021; Health Direct, 2019; Mersiades, et al., 2019; The Health Products Regulatory Authority, 2017; The Office of Drug Control, 2021)

#### 3.1.2 Brazil

Various products of industrial origin are available such as Epidiolex^®^ and Sativex^®^, and the importation of *Cannabis*-derived products is generally authorised. However, the importation of the raw plant or parts of the plant is not permitted. Products with a concentration of THC greater than 0.2% may only be prescribed when no alternative therapy is available, and the patient has reached the irreversible or terminal stage of their disease. Prescription is under the responsibility of the prescribing medical doctor. The medication may be taken either orally or by inhalation.

The cost of the treatment is generally high and is completely at the patient’s expense.

The dispensation may take place in a pharmacy, where *Cannabis* may not be processed, however.

(Crippa, et al., 2018; Marketrealist, 2019; Ministério da Saúde, 2019; Reuters, 2019; Brazilian Government, 2021)

#### 3.1.3 Canada

The situation in Canada is quite different, medical *Cannabis* (with the exception of approved industrial products) is not considered as a medicine; hence, it is not dispensed in pharmacies. Medical doctors or nurses may prescribe it for individual patients. The patient can then acquire it from a licensed vendor; grow a quantity sufficient for personal use in residence after registering with the Ministry for Health; nominate a grower in their place (a grower can only cultivate for two people); or acquire it from a provincial or area level licensed retailer. The patient is allowed to prepare *Cannabis*-based products, but the use of organic solvents such as butane, benzene, methyl-chloride, or chlorinated hydrocarbons is forbidden.

Regarding industrial products, Sativex^®^ is available for sale; it is indicated for the treatment of spasticity in multiple sclerosis. Other recommended uses include additional pain relief for neuropathic pain in adult patients with multiple sclerosis, and additional pain relief for patients with late-stage cancer who experience moderate to serious pain when already undergoing palliative care with the highest tolerable dosages of opioids. Nabilone is approved for treatment of serious nausea and vomiting associated with chemotherapy, while dronabinol is approved for the treatment of AIDS-related anorexia, and for serious nausea and vomiting associated with chemotherapy. Dronabinol was withdrawn for the Canadian market by the producer in February 2012, but not for health risks.

Generally, *Cannabis* may be used for any symptom without demonstrating the inefficacy of the previous therapies.

The approved industrial products may be reimbursed by health insurance companies, while all the others are non-reimbursable.

(Fischer, et al., 2015; Ablin, et al., 2016; Health Canada, 2016; The Health Products Regulatory Authority, 2017; Abuhasira, et al., 2018; Conseil fédéral, 2018; Government of Canada, 2019; Health Canada, 2022)

#### 3.1.4 Denmark

All medical doctors are authorised to prescribe *Cannabis*-based products as part of a 4 years pilot project launched in January 2018. As part of this project, a medical doctor may prescribe medicines that are not approved for distribution or sale in Denmark. However, the medical doctor must take full responsibility for the products they prescribe and must determine the proper dosage for each patient. Medical doctors may refer to the guidelines laid out by the Danish Medicines Agency. The imported plant products available for prescription may vary in content, but they must comply with strict standards and regulations governing the cultivation of the plant species, and the production and standardisation of the *Cannabis*-based product.

Herbal *Cannabis* is available by prescription only in pharmacies, which may also prepare magistral preparations.

Regarding industrial products, neurologists may prescribe Sativex^®^ to treat spasticity from multiple sclerosis. In general, medical doctors may prescribe imported *Cannabis*-derived medicines that have not been approved for sale in Denmark, such as Marinol^®^ and Cesamet^®^ on compassionate grounds, but only if the request is approved by the Danish Medicines Agency.

In general, the Danish Medicines Agency indicates that medical *Cannabis* be considered as a therapy only for the following conditions: painful spasticity in multiple sclerosis, painful spasticity caused by spinal cord damage, chemotherapy-induced nausea, and neuropathic pain. As part of the pilot project, *Cannabis* may, however, be prescribed to any patient even outside of the guidelines. The use of *Cannabis* is not recommended for patients under 18 years of age.

The prices of the prescribed products within the pilot project are set freely by the manufacturers. It is possible to obtain a reimbursement as of 01/01/2019 (retroactive for 2018). Patients in the terminal stages of a disease are fully reimbursed, while patients with other illnesses receive a 50% reimbursement, up to annual maximum of 10,000 Danish Krone. The reimbursement is automatically deducted at the time of the purchase in a pharmacy.

For prescriptions that are not part of the pilot project, the medical doctor may request a reimbursement for an individual patient from the Danish Medicines Agency. It will consider the request for those patients with pathologies where *Cannabis*-based treatment appears to be effective, and for those whom all other treatments with approved medicines have been used without effect.

(The Health Products Regulatory Authority, 2017; Abuhasira, et al., 2018; Krcevski-Skvarc, et al., 2018; Danish Medicines Agency, 2020; Gustavsen, et al., 2021)

#### 3.1.5 Germany

Medical doctors may prescribe medical *Cannabis* using a specific “narcotics” prescription form. The prescription may be for any condition that has no standard treatment, or the standard treatment cannot be used owing to reactions, or based on the patient’s specific condition. Among the industrial products available is Sativex^®^, which is indicated for spasticity in refractory multiple sclerosis. In addition, it is possible to prescribe dronabinol without particular restrictions regarding its indicated use. Nabilone is approved for nausea and vomiting associated with chemotherapy and unresponsive to conventional therapies. Finally, Epidiolex^®^ and many types of *Cannabis* inflorescences may also be prescribed. Magisterial preparations may be prescribed, and pharmacies may dispense extracts of *Cannabis* and inflorescences.

In the past, *Cannabis* could also be theoretically grown in residence by private individuals if conventional therapies had been inefficacious, no other alternative treatments were available, and/or to reduce the cost of therapy. Actually, this possibility has never been really applied. Since 2019, however, a system of checks on the production and supply of *Cannabis* has been introduced by the government.

The patients may request a reimbursement from health insurance companies. For this purpose the prescribing medical doctor has the task of certifying the seriousness of the disease, that the standard therapies have been ineffective, or cannot be used due to the patient’s specific condition, or that there is a reasonable likelihood that medical *Cannabis* will be effective for that subject.

(Grotenhermen and Müller-Vahl, 2012; Ablin, et al., 2016; The Health Products Regulatory Authority, 2017; Abuhasira, et al., 2018; Conseil fédéral, 2018; Federal Institute for Drugs and Medical Devices, 2018; Krcevski-Skvarc, et al., 2018; Rasche, et al., 2019; Federal Institute for Drugs and Medical Devices, 2022a; Federal Institute for Drugs and Medical Devices, 2022b; Federal Institute for Drugs and Medical Devices, 2022c; Federal Institute for Drugs and Medical Devices, 2022d; German Institute for Medical *Cannabis*, 2022)

#### 3.1.6 Israel

In Israel, patients with a prescription may use a licensed pharmacy to obtain medical *Cannabis*. There is a list of conditions for which *Cannabis* may be used, but the medical doctor may also prescribe it for other pathologies: in any case, it may only be used when other therapies have proved ineffective. The list includes neuropathic pain, serious cachexia in AIDS patients, spasticity from multiple sclerosis, pain associated with Parkinson’s disease, Tourette’s syndrome, treatment of metastatic cancer or chemotherapy-induced symptoms, inflammatory intestinal diseases and post-traumatic stress disorders.

In general, the products available are *Cannabis* inflorescences, Sativex^®^ and Epidiolex^®^. The number of medical *Cannabis* patients among the Israeli population is one of the highest in the world (on February 2022 about 100,000 Israelis -about 1% of the population-were allowed to consume medical *Cannabis*).

Sativex^®^ is recommended for spasticity from multiple sclerosis unresponsive to other treatments, or as an additional analgesic therapy in adult patients with advanced stage cancer with moderate to severe pain despite being administered the highest tolerable dosage of opioids; Epidiolex^®^ is used to treat convulsions in Dravet syndrome, and Lennox-Gastaut syndrome.

As for herbal *Cannabis*, a government-run programme produces and distributes this product. Medical *Cannabis* is supplied in two forms: as an oil extract for oral administration or sub-lingual deposition, and as the inflorescence which may be smoked or inhaled with vaporisers. The cost of the therapy is reimbursed in part by some private and state health insurance schemes.

(abcNEWS, 2022; Ablin, et al., 2016; Abuhasira, et al., 2018; Krcevski-Skvarc, et al., 2018; State of Israel - Minister of Health, 2017; State of Israel - Minister of Health, 2022; The Health Products Regulatory Authority, 2017)

#### 3.1.7 Netherlands

In Netherlands, all medical doctors may prescribe medical *Cannabis*. The pharmacies may also produce extracts using the plant material produced by the Office of Medical *Cannabis*. These are usually oil extracts to be taken orally or deposited under the tongue. Some types of inflorescences are available for this purpose: the concentration of the active molecules and granulation properties may vary. The inflorescences may also be taken in the decoction form or inhaled through vaporisers.

Sativex^®^ is approved for the treatment of spasticity from multiple sclerosis refractory to conventional therapies.

*Cannabis* is indicated for the treatment of pain (multiple sclerosis, or spinal cord injuries), chronic pain, nausea and vomiting (in chemotherapy or radiotherapy, HIV therapies, adverse reactions to hepatitis C medication), palliative care for cancer or AIDS (to increase appetite and alleviate pain, nausea and weight loss), Tourette’s syndrome, and refractory glaucoma, epilepsy and epileptic syndromes (even in children). In addition, its use is indicated in the reduction in symptomology of the following pathologies: Crohn’s disease, ulcerative colitis, itching, migraine, rheumatic conditions, ADHD, post-traumatic stress disorders, agitation in Alzheimer’s disease and cerebral trauma. Medical doctors are in any case authorised to prescribe these therapies for other conditions if they consider it fit. *Cannabis*-based products must, however, be considered only in cases where authorised medicines have inefficacious or provoked unacceptable adverse reactions.

As concerns the available herbal *Cannabis* species, Bediol^®^ (THC 6.3%; CBD 8%) is usually recommended as the first-choice therapy to alleviate pain or as an anti-inflammatory therapy. Bedrocan^®^ (THC 22%; CBD <1.0%), Bedica^®^ (THC 14%; CBD <1.0%) and Bedrobinol^®^ (THC 13.5%; CBD <1.0%) are considered more effective for the treatment of symptoms such as appetite loss, weight loss, nausea, vomiting, anorexia, cachexia, emesis, Tourette’s syndrome, and glaucoma. Bedrolite^®^ (THC <1.0%; CBD 7.5%) is employed for certain forms of epilepsy.

The healthcare system does not reimburse the cost of *Cannabis*-based medicines. In some cases, the patient may be able to claim from private insurance schemes.

(The Health Products Regulatory Authority, 2017; Abuhasira, et al., 2018; Conseil fédéral, 2018; Krcevski-Skvarc, et al., 2018; Bedrocan, 2021; Office of Medicinal Cannabis, 2022)

#### 3.1.8 Switzerland

The prescription and use of *Cannabis*-based magistral preparations is authorised for spasticity (multiple sclerosis), chronic pain, appetite loss in AIDS, and nausea, pain, and appetite loss from cancer.

The magistral preparations are prepared in a pharmacy.

Medical doctors may prescribe *Cannabis*-based medicines only after receiving authorisation from the Federal office of the Public Health System.

The cost of the therapy is not reimbursed systematically, but on a case-by-case basis.

As well as the inflorescence, it is possible to use dronabinol and Epidiolex^®^. Sativex^®^ is also authorised for use and available for treatment of spasticity from multiple sclerosis.

(Abuhasira, et al., 2018; Krcevski-Skvarc, et al., 2018; Swiss Confederation, Federal Office of Public Health, 2020; Swiss Confederation, Federal Office of Public Health, 2021a; Swiss Confederation, Federal Office of Public Health, 2021b; Swiss Confederation, Federal Office of Public Health, 2021c)

#### 3.1.9 United Kingdom

In the United Kingdom, medical *Cannabis* is generally prescribed to adults and children with rare and serious forms of epilepsy, adults suffering from nausea or vomiting from chemotherapy, and adults with muscular stiffness or spasms from multiple sclerosis. This therapy is considered only in cases in which no alternative treatment is available, or other treatments have been inefficacious. The available products are Epidiolex^®^, prescribed to patients with Lennox-Gastaut syndrome or Dravet syndrome; nabilone, which is authorised for nausea and vomiting associated with chemotherapy; dronabinol is also available, but it has no marketing authorization; and Sativex^®^, which is prescribed for muscular spasms in multiple sclerosis unresponsive to other treatments (even though it is discouraged by NICE in that it is not cost-effective).

The medical *Cannabis* therapy cannot be obtained from a general practitioner but must be prescribed by a hospital specialist registered with the General Medical Council. The medical doctor may collect data on adverse reactions, which can also be signalled directly by the patient through a yellow card system.

(Department of Health and Social Care, 2018; Medicines and healthcare products Regulatory Agency, 2020; MS Society, 2021; National Health Service, 2021; General Medical Council, 2022; National Health Service, 2022; UK Government, 2022)

#### 3.1.10 United States of America

There are significant legislative differences among the states concerning *Cannabis* in the United States. In some states the legislation in force is extremely limiting, in others significantly less restrictive. Therefore, the state laws may not be completely harmonised with federal laws.

Regarding industrial products, the FDA has approved the prescription of dronabinol and nabilone for the treatment of chemotherapy-induced nausea and vomiting. Dronabinol may also be used for the treatment of appetite and weight loss in HIV patients. Epidiolex^®^ may be prescribed for the treatment of epileptic disorders, Lennox-Gastaut syndrome and Dravet’s syndrome.

Concerning herbal *Cannabis*, only 36 states have legalised or decriminalised its use. In general, in those states which have authorised the use of medical use *Cannabis*, there are restrictions on its prescription. Depending to the local laws, therefore, *Cannabis* may be prescribed for pain, anxiety, epilepsy, glaucoma, appetite and weight loss associated with AIDS, inflammatory intestinal disturbances irritable intestine syndrome, motor disturbances due to Tourette’s syndrome or multiple sclerosis, nausea and vomiting caused by chemotherapy, sleep disorders, posttraumatic stress disorders. Some states allow the addition, at the prescribing medical doctor’s discretion, of pathologies other than those expressly stated.

Generally, medical doctors do not need specific training to prescribe *Cannabis*, but in many states, it is necessary to register before doing so. In other states, medical doctors must attend a short training course to be able to register. In some states, it is enough that the medical doctor gives advice verbally to take medical *Cannabis*, or its use may be recommended by a health care professional who is not a medical doctor. On the other hand, in some states, it is necessary that two medical doctors confirm the need for a *Cannabis*-based treatment for a patient. Depending on the state, *Cannabis* may be supplied to the patient by licensed dispensaries, or it may be grown at home by the patient or by a caregiver.

Smoking medical *Cannabis* is prohibited in some states. Similarly even the edible forms are prohibited in some states. Generally, the administration is performed orally or by vaporiser.

Patients are generally registered so that the possession and use of medical *Cannabis* is not prosecuted.

Abuhasira, et al., 2018; Alharbi, 2020; Carliner, et al., 2017; Choo and Emery, 2017; Corroon and Kight, 2018; Johnson, et al., 2021; Mead, 2017; National Conferences of State Legislatures, 2022; ProCon, 2022; Ryan, et al., 2021; The Health Products Regulatory Authority, 2017)

### 3.2 Study Protocols and Clinical Trials

There are 43 publications of proposed, or executed, clinical trial protocols in those countries whose legislation has been analysed; eight of these regarded proposed clinical trial protocols.

Hence, 35 publications regarded actual clinical trial data. These were sub-divided into three groups: the first, “positive outcome,” included those studies which demonstrated the efficacy of the preparation administered, or that the actual results were in line with those expected (18). The second group, “negative outcome,” included those studies where the authors reported that the administered product was no more efficacious than the placebo (5). Finally, the third group, “inconclusive outcome,” comprised those studies where the results were not conclusive (12).

The characteristics of the taken into account clinical studies are summarized in Table 1.

**TABLE 1:** Characteristics of the selected clinical trials.

Clinical trials with a POSITIVE outcome							
Country		Study TITLE	Study type	Administration	Product	Condition	Number of patients
1	AUSTRALIA	A pilot randomised placebo-controlled trial of cannabidiol to reduce severe behavioural problems in children and adolescents with intellectual disability Efron et al. (2021)	Double-blinded Placebo-controlled Randomized	Oral (solution)	CBD	Severe behavioural problems (in children and adolescents with intellectual disability)	8
2	AUSTRALIA	Oral THC:CBD cannabis extract for refractory chemotherapy-induced nausea and vomiting: a randomised, placebo-controlled, phase II crossover trial Grimison et al. (2020)	Double-blinded Multicentre Placebo-controlled Randomized	Oral (capsules)	Herbal *Cannabis*: THC:CBD (1:1 ratio) *Cannabis sativa* L. extract	Refractory chemotherapy-induced nausea and vomiting	80 enrolled 72 completed the study
3	AUSTRALIA	Cannabis use in patients 3 months after ceasing nabiximols for the treatment of cannabis dependence: Results from a placebo-controlled randomised trial Lintzeris et al. (2020)	Double-blinded Multicentre Placebo-controlled Randomized	Oro-mucosal spray	Sativex^®^ (obtained from *Cannabis sativa* L.)	*Cannabis* dependence	128
4	AUSTRALIA	A Phase 1, Randomised, Placebo-Controlled, Dose Escalation Study to Investigate the Safety, Tolerability and Pharmacokinetics of Cannabidiol in Fed Healthy Volunteers Perkins et al. (2020)	Double-blinded Placebo-controlled Randomized	Oral (solution)	CBD	Safety, tolerability and pharmacokinetics evaluations on healthy volunteers	24
5	ISRAEL	The pharmacokinetics, efficacy, and safety of a novel selective dose cannabis inhaler in patients with chronic pain: A randomized, double-blinded, placebo-controlled trial Almog et al. (2020)	Double-blinded Placebo-controlled Randomized	Inhalation	Herbal *Cannabis*: Bedrocan^®^ (22% THC, <1% CBD)	Chronic pain	27 enrolled 25 completed the study
6	ISRAEL	Pharmacokinetic investigation of synthetic cannabidiol oral formulations in healthy volunteers Izgelov et al. (2020)	Blinded	Oral (powder, oil or self-nano-emulsifying drug delivery system)	CBD	Oral absorption processes of synthetic CBD when given in different oral formulations in healthy volunteers	12
7	ISRAEL	The safety, tolerability, and effectiveness of PTL-101, an oral cannabidiol formulation, in pediatric intractable epilepsy: A phase II, open-label, single-center study Mitelpunkt et al. (2019)	Open-label	Oral (capsules)	CBD	Treatment-resistant epilepsy (in paediatric patients)	16 enrolled 11 completed
8	ISRAEL	Effect of adding medical cannabis to analgesic treatment in patients with low back pain related to fibromyalgia: an observational cross-over single centre study Yassin et al. (2019)	Observational	Inhalation	THC:CBD (1:4 ratio) herbal *Cannabis*	Low back pain related to fibromyalgia	31
9	SWITZERLAND	Cannabidiol enhances verbal episodic memory in healthy young participants: A randomized clinical trial Hotz et al. (2021)	Double-blinded Placebo-controlled Randomized	Inhalation	CBD	Verbal episodic memory in healthy young subjects	39
10	UNITED KINGDOM	Cannabidiol for the treatment of cannabis use disorder: a phase 2a, double-blind, placebo-controlled, randomised, adaptive Bayesian trial Freeman et al. (2020)	Double-blinded Placebo-controlled Randomized	Oral (capsules)	CBD	Desire to stop using *Cannabis*	82
11	UNITED KINGDOM	Normalization of mediotemporal and prefrontal activity, and mediotemporal-striatal connectivity, may underlie antipsychotic effects of cannabidiol in psychosis O'Neill et al. (2021)	Double-blinded Placebo-controlled Randomized	Oral (capsules)	CBD	Psychosis	34 enrolled 32 completed
12	UNITED KINGDOM	Effects of cannabidiol on brain excitation and inhibition systems; a randomised placebo-controlled single dose trial during magnetic resonance spectroscopy in adults with and without autism spectrum disorder Pretzsch et al. (2019a)	Double-blinded Placebo-controlled Randomized	Oral (solution)	CBD	Brain excitation and inhibition systems	34
13	UNITED KINGDOM	The effect of cannabidiol (CBD) on low-frequency activity and functional connectivity in the brain of adults with and without autism spectrum disorder (ASD) Pretzsch et al. (2019c)	Double-blinded Placebo-controlled Randomized	Oral (solution)	CBD	Low-frequency activity and functional connectivity in the brain	34
14	UNITED KINGDOM	Dissociable effects of cannabis with and without cannabidiol on the human brain’s resting-state functional connectivity Wall et al. (2019)	Double-blinded Placebo-controlled Randomized	Inhalation	Herbal *Cannabis*: *Cannabis* containing both THC and CBD; *Cannabis* containing THC	Human brain’s resting-state networks	17
15	United States	Food effect on pharmacokinetics of cannabidiol oral capsules in adult patients with refractory epilepsy Birnbaum et al. (2019)	Open-label	Oral (capsules)	CBD	Refractory epilepsy	11 enrolled 8 completed the study
16	United States	Cannabidiol for the Reduction of Cue-Induced Craving and Anxiety in Drug-Abstinent Individuals With Heroin Use Disorder: A Double-Blind Randomized Placebo-Controlled Trial Hurd et al. (2019)	Double-blinded Placebo-controlled Randomized	Oral (solution)	CBD	Drug cue–induced craving and anxiety in drug-abstinent individuals with heroin use disorder	50 enrolled 42 completed
17	United States	The Effectiveness of Topical Cannabidiol Oil in Symptomatic Relief of Peripheral Neuropathy of the Lower extremities Xu et al. (2020)	Double-blinded Placebo-controlled Randomized	Topical (cream)	CBD	Peripheral neuropathy	29
18	United States	A randomized trial of medical cannabis in patients with stage IV cancers to assess feasibility, dose requirements, impact on pain and opioid use, safety, and overall patient satisfaction Zylla et al. (2021)	Placebo-controlled Randomized	Inhalation, oral, topical	A variety of formulations (also based on herbal *Cannabis*) with differing ratios of THC/CBD were provided depending on individual patient symptoms	Pain, opioid use, safety, and satisfaction in cancer patient	30
**Clinical Trials With A NEGATIVE Outcome**							
**Country**		**Title**	**Study Type**	**Administration**	**Administerd Product**	**Condition**	**Number Of Patients**
1	AUSTRALIA	The CANBACK trial: a randomised, controlled clinical trial of oral cannabidiol for people presenting to the emergency department with acute low back pain Bebee et al. (2021)	Double-blinded Placebo-controlled Randomized	Oral (solution)	CBD	Low back pain	100
2	BRAZIL	Cannabidiol for COVID-19 Patients with Mild to Moderate Symptoms (CANDIDATE Study): A Randomized, Double-Blind, Placebo-Controlled Clinical Trial Crippa et al. (2021)	Double-blinded Placebo-controlled Randomized	Oral (solution)	CBD	COVID-19	105
3	UNITED KINGDOM	The acute effects of cannabidiol on the neural correlates of reward anticipation and feedback in healthy volunteers Lawn et al. (2020)	Double-blinded Placebo-controlled Randomized	Oral (capsules)	CBD	Neural correlates of reward anticipation and feedback	28 enrolled 24 completed the study
4	UNITED STATES	The short-term impact of 3 smoked cannabis preparations versus placebo on PTSD symptoms: A randomized cross-over clinical trial Bonn-Miller et al. (2021)	Double-blinded Placebo-controlled Randomized	Inhalation	Herbal *Cannabis*: *Cannabis* containing 12% THC and <0.05% CBD; *Cannabis* containing 11% CBD and 0.50% THC; *Cannabis* containing 7.9% THC and 8.1% CBD	Posttraumatic Stress Disorder	80 enroled 76 completed stage I 74 completed stage II
5	UNITED STATES	Acute effects of cannabinoids on symptoms of obsessive-compulsive disorder: A human laboratory study Kayser et al. (2020)	Double-blinded Placebo-controlled Randomized	Inhalation	Herbal *Cannabis*: *Cannabis* containing THC 7% and CBD 0.18%; *Cannabis* containing 0.4% THC and 10.4% CBD	Obsessive-compulsive disorder	14 enrolled 12 completed the study

**Clinical Trials With an INCONCLUSIVE Outcome**							
**Country**		**Title**	**Study Type**	**Administration**	**Administerd Product**	**Condition**	**Number of patients**
1	AUSTRALIA	Citalopram and Cannabidiol: *In Vitro* and *In Vivo* Evidence of Pharmacokinetic Interactions Relevant to the Treatment of Anxiety Disorders in Young People Anderson et al. (2021)	Controlled Randomized	Oral (capsules)	CBD	Anxiety disorders	6
2	AUSTRALIA	Model-based analysis on systemic availability of co-administered cannabinoids after controlled vaporised administration Liu et al. (2020)	Double-blinded Placebo-controlled Randomized	Inhalation	THC; CBD; THC + low-dose CBD; THC + high-dose CBD	Evaluate the active dosage	36
3	AUSTRALIA	A randomised controlled trial of vaporised Δ9-tetrahydrocannabinol and cannabidiol alone and in combination in frequent and infrequent cannabis users: acute intoxication effects Solowij et al. (2019)	Double-blinded Placebo-controlled Randomized	Inhalation	THC; CBD; THC + low-dose CBD; THC + high-dose CBD	Evaluate the acute intoxication effects	36
4	ISRAEL	Oral CBD-rich Cannabis Induces Clinical but Not Endoscopic Response in Patients with Crohn’s Disease, a Randomised Controlled Trial Naftali et al. (2021a)	Double-blinded Placebo-controlled Randomized	Oral (solution)	THC:CBD (1:4 ratio) herbal *Cannabis*	Crohn’s Disease	56
5	ISRAEL	Cannabis is associated with clinical but not endoscopic remission in ulcerative colitis: A randomized controlled trial Naftali et al. (2021b)	Double-blinded Placebo-controlled Randomized	Inhalation	Herbal *Cannabis*: *Cannabis* containing 16% THC and 0.1% CBD	Ulcerative colitis	32
6	ISRAEL	Cannabinoid treatment for autism: a proof-of-concept randomized trial Aran et al. (2021)	Double-blinded Placebo-controlled Randomized	Oral (solution)	THC:CBD (1:20 ratio) herbal *Cannabis;* purified CBD: THC (1:20 ratio)	Autism spectrum disorder in children and adolescents	150
7	NETHERLANDS	An experimental randomized study on the analgesic effects of pharmaceutical-grade cannabis in chronic pain patients with fibromyalgia Van de Donk et al. (2019)	Double-blinded Placebo-controlled Randomized	Inhalation	Herbal *Cannabis*: Bedrocan^®^ (22% THC, < 1% CBD); Bediol^®^ (6.3% THC, 8% CBD); Bedrolite^®^ (9% CBD, <1% THC)	Pain in patients with fibromyalgia	20
8	UNITED KINGDOM	Effects of cannabidivarin (CBDV) on brain excitation and inhibition systems in adults with and without Autism Spectrum Disorder (ASD): a single dose trial during magnetic resonance spectroscopy Pretzsch et al. (2019b)	Double-blinded Placebo-controlled Randomized	Oral (solution)	Cannabidivarin	Autism Spectrum Disorder	34
9	UNITED STATES	Effect of Inhaled Cannabis for Pain in Adults With Sickle Cell Disease. A Randomized Clinical Trial Abrams et al. (2020)	Double-blinded Placebo-controlled Randomized	Inhalation	Herbal *Cannabis*: *Cannabis* containing 4.4% THC and 4.9% CBD	Pain in patients with sickle cell disease	23
10	UNITED STATES	Effects of oral, smoked, and vaporized cannabis on endocrine pathways related to appetite and metabolism: a randomized, double-blind, placebo-controlled, human laboratory study Farokhnia et al. (2020)	Double-blinded Placebo-controlled Randomized	Inhalation and oral (food)	THC	Appetite and metabolism in healthy valuntaires	20
11	UNITED STATES	Effects of Hemp Extract on Markers of Wellness, Stress Resilience, Recovery and Clinical Biomarkers of Safety in Overweight, But Otherwise Healthy Subjects Lopez et al. (2020)	Double-blinded Placebo-controlled Randomized	Oral (solution)	CBD (from Herbal *Cannabis* oil extract)	Markers of wellness, stress resilience, recovery and clinical biomarkers of safety in overweight, but otherwise healthy subjects	65
12	UNITED STATES	Cognitive function and adaptive skills after a 1-year trial of cannabidiol (CBD) in a pediatric sample with treatment-resistant epilepsy Thompson et al. (2020)	Open-label	Oral (solution)	CBD	Cognitive function and adaptive skills in a paediatric patients with treatment-resistant epilepsy	38

#### 3.2.1 Clinical Trials With a Positive Outcome

Of the 18 studies in this category, 4 were conducted in Australia, 4 in Israel, 1 in Switzerland, 5 in the United Kingdom, and 4 in the United States.

Regarding the study design, 2 were multi-centred, 13 used the double-blind method, 14 had a randomised control design, and 14 used a placebo control group.

The sample size varied greatly, from a minimum of 8 to a maximum of 128 enrolled subjects.

As for the products used in the trials, 12 studies administer CBD, 6 studied herbal *Cannabis* derivatives.

CBD was administered orally in 10 cases, topically and by inhalation in only one study. The herbal *Cannabis* derivatives were administered by inhalation in 3 cases, and by the oral route in 2 cases. One study considered products to be administered orally, by inhalation or topically.

In 9 studies, the *Cannabis* derivatives were administered in addition to a standard therapy.

The most commonly studied conditions were behaviour, cerebral activity, and memory (6), pain (4), addiction or abstinence to drugs (3), epilepsy (2), pharmacokinetic studies, safety, and tolerability (2), and nausea and vomiting (1). Two studies were carried out on a paediatric population.

In general, the studies involving the administration of CBD regarded epilepsy, addiction or abstinence to drugs, behaviour, cerebral activity and memory, peripheral neuropathy, pharmacokinetic studies, and safety and tolerability.

Instead, studies administering herbal *Cannabis* derivatives focused mainly about pain and then about nausea and vomiting, cerebral activity and *Cannabis* dependence. In most cases both THC and CBD were administered in different ratios. In some cases, a herbal *Cannabis* strain was used with a high concentration of THC.

(Almog, et al., 2020; Birnbaum, et al., 2019; Efron, et al., 2021; Freeman, et al., 2020; Grimison, et al., 2020; Hotz, et al., 2021; Hurd, et al., 2019; Izgelov, et al., 2020; Lintzeris, et al., 2020; Mitelpunkt, et al., 2019; O'Neill, et al., 2021; Perkins, et al., 2020; Pretzsch, et al., 2019a; Pretzsch, et al., 2019c; Wall, et al., 2019; Xu, et al., 2020; Yassin, et al., 2019; Zylla, et al., 2021)

#### 3.2.2 Clinical Trials With a Negative Outcome

Five trials had a negative outcome. Two of these were conducted in the United States, 1 in Australia, 1 in Brazil and 1 in the United Kingdom.

All of the trials had a randomised control, used a placebo control group, and a double-blind control. The sample size ranged from 14 to 105 enrolled subjects.

As for the products used, 3 studies administered oral preparations containing CBD. 2 studies were based on the administration of inflorescences by inhalation. 4 studies out of 5 administered the product in addition to a standard therapy.

The conditions studied in these trials with CBD were pain, COVID-19 infection, and the effects on neural correlates of reward anticipation and feedback. Herbal *Cannabis*, in three different forms and different ratios of THC/CBD), was administered to evaluate its efficacy in the treatment of Obsessive-Compulsive Disorder (OCD) and Post-Traumatic Stress Disorder (PTSD).

None of these studies demonstrated that the administered product was more efficacious than the placebo control.

(Kayser, et al., 2020; Lawn, et al., 2020; Bebee, et al., 2021; Bonn-Miller, et al., 2021; Crippa, et al., 2021)

#### 3.2.3 Clinical Trials With an Inconclusive Outcome

12 studies had an inconclusive outcome: 3 were conducted in Australia, 3 in Israel, 1 in the Netherlands, 1 in the United Kingdom and 4 in the United States.

Regarding study design, 10 included a double-blind system, 11 had a randomised control, and 10 utilised a placebo control group. The sample size ranged from a minimum of 6 subjects to a maximum of 150 individuals. Two of the studies were conducted on paediatric subjects.

Concerning the products used, 2 studies administered CBD alone, one study used THC alone, 1 study administered cannabidivarin, 2 studies administered THC and CBD, both alone and in a mixture, 5 studies administered herbal *Cannabis* derivatives, and 1 study administered both THC and CBD as well as a herbal *Cannabis* extract.

CBD and cannabidivarin were administered orally; THC, and the mixtures of THC and CBD were administered by inhalation. THC was also administered orally. The herbal *Cannabis* derivatives were administered by inhalation in 3 studies, while they were for oral use in 2 studies. 1 study used oral administration of a herbal *Cannabis* extract or an equivalent mixture of THC and CBD.

Six trials predicted that the administration was additional to standard therapy.

The conditions to be studied for the efficacy of CBD were anxiety and cognitive function in patients suffering from epilepsy. THC and/or CBD were administered to evaluate the active dosage or to study its effects on problems linked to appetite and metabolism, herbal *Cannabis* derivatives were studied to evaluate their activity in Crohn’s disease, ulcerative colitis, pain, haemolytic anaemia, markers of wellness and clinical biomarkers in obese patients. Trials related to autism were conducted with, as well as cannabidivarin, the administration of a herbal *Cannabis* extract or an equivalent mixture of THC and CBD.

When herbal *Cannabis* derivatives were administered, the concentration of THC and CBD, and the ratio of the two varied greatly among the trials. Some used products with a high concentration of THC, while others used products with a high concentration of CBD. In 1 trial, different types of inflorescences were administered to evaluate the most efficacious ratio of THC to CBD concentrations against pain.

(Pretzsch, et al., 2019b; Solowij, et al., 2019; Van de Donk, et al., 2019; Abrams, et al., 2020; Farokhnia, et al., 2020; Liu, et al., 2020; Lopez, et al., 2020; Thompson, et al., 2020; Naftali, et al., 2021a; Anderson, et al., 2021; Aran, et al., 2021; Naftali, et al., 2021b)

#### 3.2.4 Study Protocols

There are 8 examples of published protocols that have not yet initiated the clinical trial phase. 4 are in Australia, and 1 each in Denmark, Canada, Germany, and Netherlands. The number of enrolled subjects is between 10 and 180 in total. One study will be carried out among the paediatric population.

Concerning the study design, 3 will be multi-centre studies, 7 use a double-blind system, 8 are randomised, and 7 use a placebo control group.

Regarding the products to be used, 4 protocols will use the oral administration of THC and CBD. The ratio between the components in question varies from study to study. In 2 protocols, the administration of CBD is also foreseen. One protocol foresees the administration of both CBD and a preparation containing a high concentration of THC.

For those studies using THC and CBD mixtures, the pathologies to be studied are, pain, dementia, spasms, and the activation of the immune system in HIV patients. Instead, the CBD alone preparations will be administered for behavioural problems and phobias. The herbal-*Cannabis* derived product will be administered for chronic tic disorder. The protocol that foresees the administration of both CBD and a preparation with a high concentration of THC will focus on the alleviation of pain.

(Costiniuk, et al., 2019; Hendricks, et al., 2019; Urbi, et al., 2019; Van der Flier, et al., 2019; Efron, et al., 2020; Hardy, et al., 2020; Jakubovski, et al., 2020; Timler, et al., 2020)

## 4 Discussion

From the analysis of the current legislation in states where clinical trials and proposed protocols on medical *Cannabis* and derived products have been published in the last 3 years, many significant differences have been found regarding the products available, the indicated pathologies for which it may be prescribed, the production of the raw plant material, as well as its reimbursement and prescription. It was evaluated to consider the studies published in the last 3 years supposing that the researchers have benefited from the latest knowledge on medical *Cannabis* and to make an overview of the pathologies currently under study.

In particular, regarding industrial products, practically every country, with the exception of the United States, has approved the use of Sativex^®^. However, Epidiolex^®^, dronabinol.Netherlands, and nabilone are also quite common.

In all the countries, the use of herbal *Cannabis* is also authorised. The only exception is Brazil, which is certainly the country with the most restrictive legislation. Netherlands is the only country to provide directions for use, which are not binding, but quite strict, regarding the plant strain to be used for a determined pathology based on the concentration of active molecules (THC and CBD). Instead, for the other countries, it must be pointed out that the current legislation provides for the use of inflorescences or herbal *Cannabis* extracts without providing specific directions concerning the recommended concentration of active molecules to treat a determined condition.

Regarding the pathologies or symptoms associated with the more or less well-defined conditions, the most common are pain, nausea, vomiting, spasticity, and epilepsy followed by spasms, and weight and appetite loss. The less frequently indicated conditions in this case include Tourette’s syndrome, PTSD, and glaucoma. In many countries, additional conditions are considered in more or less detail.

In this regard, it is interesting to note that the country with the greatest number of specifically recommended pathologies not indicated in other countries is the Netherlands: perhaps based on the longstanding use of *Cannabis* both for medical use and recreational purposes. Although the legislation regarding medical *Cannabis* is quite comprehensive in all the countries considered, some of them, namely Australia, Canada, Denmark, Germany, Israel, Netherlands, and the United States, also permit the prescription of *Cannabis* for any therapeutic application at the discretion of the medical doctor. However, in Germany, Netherlands and Israel, this is limited to cases in which other therapies have proved ineffective, excessive adverse reactions to standard treatments have occurred, or valid alternative treatments are not available. Instead, in Australia, Canada, Denmark, and the United States, therapeutic strategies different from those specified are authorised regardless of any prior treatment. The prescription of medical *Cannabis* for any condition certainly does not conform to the procedures generally in force for other medicinal products, and especially products with a psychoactive effect such as those prepared containing THC.

It is interesting to note that in Canada, and in some states in the United States, the medical inflorescences may be grown directly by the patient, and the treatment may be recommended by a health worker, and not only a medical doctor; in the event that the plant species is not home-grown, it is distributed through a licensed dispensary. In Germany, Israel and Netherlands, herbal *Cannabis* is grown locally under the supervision of a government agency. This is significant if one considers that, in these three countries, the prescription process is highly deregulated regarding the recommended pathologies to be treated with *Cannabis*, but the same does not apply to its cultivation.

The normal administration routes are oral or by inhalation. Some countries, such as Israel, authorise smoking *Cannabis* inflorescences as a route of administration, something that is categorically banned in some states of the United States.

In addition, regarding prescription, it is noteworthy that the United Kingdom is the only country where this must be obtained from a hospital specialist. In some states in the United States, on the other hand, the prescribing medical doctor must be registered to prescribe this therapy and have attended a specific training course. In Australia and Switzerland, medical doctors may write the prescription only after receiving authorisation from a specific agency. Therefore, there is a different focus on the prescription process and hence inhomogeneity in this aspect too. The treatment costs are generally borne by the patient, and no reimbursement is foreseen, unless it is from a private health insurance scheme. This certainly restricts access to this kind of therapy to the more privileged members of society.

Concerning the results of the clinical trials, some interesting observations may be made. In the first place, a greater number of studies have been published in certain countries. These countries are the United States (11) and Australia (9), followed by Israel (7) and the United Kingdom (7). In general, the majority of the studies featured randomisation, the use of a double-blind method, and a placebo control group: these are factors which guarantee the quality of the data gathered. On the other hand, the majority of the studies took place with a small sample size. Moreover, the studies made use of a heterogeneous population: healthy and ill volunteers, adults and children, acute and chronically ill patients, and subjects who had previously used or had never used *Cannabis* prior to the study. Factors that, being so numerous, make it particularly challenging to draw any conclusive evaluations of the results of these trials, and more in general, the real efficacy of medical *Cannabis*.

Considering only the studies with a positive outcome, it should be noted that the studied pathologies are coherent with those provided for in current legislation i.e., pain, epilepsy, nausea, and vomiting; on the contrary, psychosis, behavioural problems, memory and cerebral activity represent a novelty. Furthermore, there is a net distinction between the products used based on the different conditions to be treated: the trials on pain, nausea and vomiting with positive outcomes administered herbal *Cannabis* derivatives in which, in 3 cases out of 4, both THC and CBD are present; the other studies with a positive outcome administered CBD alone. In those trials with a negative outcome, CBD was administered for pain, while herbal *Cannabis* derivatives were used for conditions such as OCD or PTSD. This consideration supports the use of herbal *Cannabis* in which both THC and CBD are present for pain, even though it should be stressed that the studies with a positive outcome for this pathology had a maximum of 30 enrolled subjects.

The studies with an inconclusive outcome regarded a variegated list of conditions including anxiety, Crohn’s syndrome, ulcerative colitis, pain, and appetite loss. Many of these are already included in some national regulations although the efficacy of *Cannabis* in these cases according to the currently available data is not satisfactorily demonstrated.

It is evident that the only pathology present in all three study categories is pain, for which 4 studies had a positive outcome, 1 had a negative outcome, and 1 had an inconclusive outcome.

Among the study protocols to be trialled, pain and spasticity appear again, approved by legislation in most countries and the object of numerous studies, as well as a number of less-investigated conditions such as dementia, phobias, tic disorders and the activation of the immune system in HIV patients.

Based on the research conducted, it is, therefore, possible to stress that in spite of the growing number of recent studies on medical *Cannabis*, many of which have had a positive outcome while many others have had an inconclusive or negative outcome. The presumed broad spectrum action of *Cannabis* has led to the initiation of many trials and the preparation of many study protocols for a wide range of pathologies with the enrolment of subjects with diverse characteristics from study to study. This means that there is very little data for each pathology or symptomology.

Another important factor is that the products used are very diverse from each other; consequently, a comparison is extremely difficult to make, especially for the herbal products. All of the trials indicate the precise dosages used in terms of active molecules, but when it comes to inflorescences, or extracts derived from them, the concentration is provided only for the THC and CBD content and not for the other active molecules. Furthermore, the diverse administration routes make a comparison based on pharmacokinetics difficult for the molecules of interest.

Therefore, it is difficult to compare the studies and draw conclusions concerning the efficacy of the protocol for the single pathologies. However, for some, substantial evidence is emerging regarding their efficacy and the suitable products to ensure that. From the analysed data, it is clear that the best pain treatment is herbal *Cannabis* derivatives containing both THC and CBD, just as the best way to treat epilepsy is to administer CBD.

One interesting point is that for some of the pathologies approved for treatment with medical *Cannabis* under the current legislation, the data do not paint a definitive picture. This is true for conditions such as anxiety, ulcerative colitis, Crohn’s syndrome, and appetite enhancement.

On the other hand, the current legislation often authorises inflorescences or extracts without indicating the exact concentration of the active molecules. In parallel, many studies use different plant strains or study a small number of subjects, making it difficult to compare and consequently interpret the results. Moreover, in many studies, the *Cannabis*-based medicines were administered in addition to other treatments making any evaluation of their efficacy it even more complex.

## 5 Conclusion

Medical *Cannabis* is often considered as if it were a single active component, but, in fact, there are countless possible variations. Hence, it will be some time before the current list of pathologies that each product may be used for can be updated based on definitive clinical data on the efficacy of the various components. Certainly, the development of standardised industrial products will facilitate the execution of more meaningful trials compared to those that involve the administration of inflorescences or derived extracts prepared using a variety of methods and, thus, highly variable in terms of concentration of the active molecules.

The authors want moreover to put in evidence that, despite legislation authorising the use of medical *Cannabis* and instituting the national production centre for inflorescences more than 5 years ago, Italy is still among the states where clinical trials have not been conducted. This gap is due to legal restrictions on the approval and conduction of clinical trials in this field, and the difficulty in sourcing the raw plant material, of which there is always a shortage. The result of this is therapies using inflorescences and extracts which have never undergone specific clinical trialling.

In the end, the influence of the media, economic interests, and the demands of associations representing patients affected by these diseases and conditions, for whom *Cannabis* is a panacea, means that in many countries it is currently possible to use medical *Cannabis* even though the scientific data do not entirely support the signs of efficacy: certainly this is a special case where the consolidated procedures for the administration of any product in the medical field have been either overlooked or ignored. It is time that the regulatory agencies considered whether this is actually safeguarding the health of patients.

## Limits

The analysis of the current legislation may not be exhaustive in that it refers only to public texts available online.

## Data Availability

The original contributions presented in the study are included in the article/Supplementary Material, further inquiries can be directed to the corresponding author.

## Footnotes

1 Epidiolex^®^ has received approval in the European Union under the tradename Epidyolex^®^.

## Abbreviations

ADHD, Attention-Deficit/Hyperactivity Disorder; AIDS, Acquired ImmunoDeficiency Syndrome; CBD, CannaBiDiol; COVID-19, COronaVIrus Disease 2019; FDA, Food and Drug Administration; HIV, Human Immunodeficiency Virus; NICE, National Institute for health and Care Excellence; OCD, Obsessive-Compulsive Disorder; PTSD, Post-Traumatic Stress Disorder; THC, delta-9-TetraHydroCannabinol; United States, United States of America.
